# Lateral pharyngeal approach in surgery for malignant tumors of the posterior wall of the hypopharynx

**DOI:** 10.1097/MD.0000000000046695

**Published:** 2025-12-19

**Authors:** Qingyong Chen, Xiaoli Liu, Zhipeng Chen, Qiang Shao, Huaiqing Lv, Liqiang Lin

**Affiliations:** a Linyi People’s Hospital Affiliated to Shandong Second Medical University, Otolaryngology Head and Neck Surgery, Linyi, China; b School of Public Health, Shandong Second Medical University, Weifang, China; c Lanshan District Yitang Center Hospital, Internal Medicine, Linyi, China.

**Keywords:** lateral pharyngeal approach, posterior hypopharyngeal wall cancer, surgical treatment

## Abstract

This study aims to evaluate the therapeutic efficacy and clinical application value of the lateral pharyngeal approach for surgical resection of posterior hypopharyngeal wall cancer. A retrospective analysis was conducted on 40 patients who underwent surgical resection of malignant tumors of the posterior hypopharyngeal wall via the lateral pharyngeal approach in the Department of Otorhinolaryngology-Head and Neck Surgery at the People’s Hospital of Linyi between January 2019 and January 2023. The cohort included 36 males and 4 females, aged 46 to 78 years, with a median age of 63 years. Preoperative TNM staging (T1–T3) was confirmed through electronic laryngoscopy, pathological biopsy, and CT or MRI imaging. Tumor resection was performed entirely via the lateral pharyngeal approach. Pharyngeal cavity defects were repaired by mobilizing the submandibular gland, and postoperative follow-up evaluations recorded swallowing function, respiratory function, complications, and recurrence. After a follow-up period of 12 to 48 months, with an average follow-up of 28 months, 40 patients successfully had their tubes removed. Their swallowing and breathing functions were good, and they were able to eat and breathe normally. Up to now, there was no serious complications such as pharyngocutaneous fistula, bleeding, wound infection, tumor recurrence, and death. The lateral pharyngeal approach for resecting posterior hypopharyngeal wall cancer enables complete tumor excision with minimal trauma, limited tissue loss, and a low complication rate. These outstanding results were obtained in a highly selected T1–T3 cohort treated by a single surgical team; they should be regarded as promising preliminary findings that mandate validation through larger, multi-center prospective studies before the technique is widely adopted.

## 1. Introduction

Hypopharyngeal carcinoma is a common malignant tumor in otorhinolaryngology and head and neck surgery, accounting for approximately 5% of head and neck malignancies.^[1]^ Based on the site of occurrence, hypopharyngeal carcinoma can be classified into pyriform fossa carcinoma, posterior cricoid mucosa carcinoma, and posterior hypopharyngeal wall carcinoma. The posterior hypopharyngeal wall region lies between the plane of the epiglottis tip and the cricopharyngeal muscle, covering the posterior hypopharyngeal wall anterior to the vertebrae. Posterior wall carcinoma of the hypopharynx is rare in clinical practice. Its hidden location, lack of obvious early symptoms, invasive growth pattern, and propensity for submucosal infiltration and diffusion contribute to early lymph node involvement and distant metastasis. These factors make it one of the head and neck malignancies with an exceptionally poor prognosis.^[2]^ In recent years, induction chemotherapy, concurrent radiotherapy, targeted therapy, and immunotherapy have been widely employed in the comprehensive treatment of hypopharyngeal cancer. However, large-scale studies indicate that these approaches have not significantly improved overall survival rates. Instead, surgery-led comprehensive treatment plans have demonstrated superiority in achieving better local control and progression-free survival rates.^[3]^ Due to the proximity of posterior hypopharyngeal wall carcinoma to the spinal cord, radiotherapy doses are often limited, leading to suboptimal treatment outcomes. As a result, surgery remains a critical treatment modality.^[4]^ The hypopharyngeal mucosa, characterized by its highly folded or convoluted structure, plays a vital role in essential physiological functions such as respiration and swallowing. Choosing the appropriate surgical approach and reconstruction techniques is not only crucial for the prognosis but also essential for preserving the residual function of the pharynx and larynx while minimizing postoperative sequelae. This study conducted a retrospective analysis of 40 patients with T1-T3 stage posterior pharyngeal wall cancer who underwent pharyngeal lateral approach surgery and submandibular gland repair to restore the pharyngeal cavity in our department. The treatment experience was summarized for clinical reference. The results are reported as follows.

## 2. Research methods and materials

### 2.1. Clinical data

Forty patients who underwent resection of malignant tumors of the hypopharyngeal posterior wall via the lateral pharyngeal approach at the Department of Otolaryngology-Head and Neck Surgery, Linyi People’s Hospital, from January 2019 to January 2023 were selected for this study. The cohort included 36 men and 4 women, aged 46 to 78 years, with a median age of 63 years. All patients had hypopharyngeal posterior wall cancers, diagnosed based on preoperative electron laryngoscopy, biopsy, and CT or MRI imaging, with TNM staging of T1–T3. The inclusion criteria were as follows: patients with T1–T3 malignant tumors of the hypopharyngeal posterior wall, diagnosed by preoperative electron laryngoscopy, biopsy, and imaging examinations; no prior treatments; intraoperative frozen section and postoperative histopathological confirmation of squamous cell carcinoma of the hypopharyngeal posterior wall. Exclusion criteria included: secondary hypopharyngeal cancer; congenital malformations of the hypopharynx or larynx; patients unable to tolerate surgical treatment; and patients lost to follow-up. TNM staging was performed for all patients according to the 2018 8th edition of the AJCC^[5]^ staging criteria: 3 cases of T1N0M0, 8 cases of T2N0M0, 14 cases of T3N0M0, and 15 cases of T3N1M0. The study was conducted in accordance with the Declaration of Helsinki on ethical principles for medical research and was approved by the Science and Technology Ethics Committee of Linyi People’s Hospital (Approval Number: YX200740). All patients and their families provided informed consent, and all surgeries were performed by the same senior physician.

### 2.2. Surgical approach

**Figure 1.**
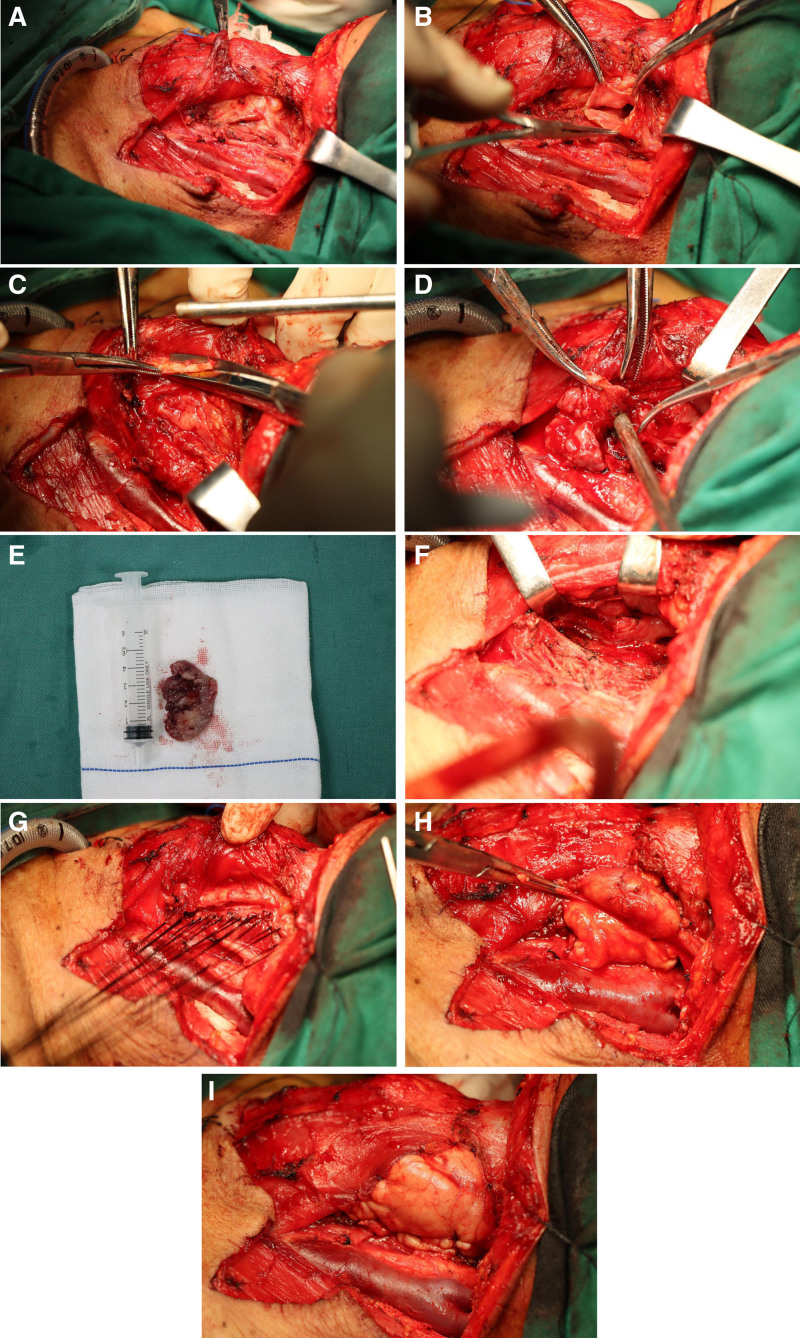
(A) View of the exposed affected thyroid cartilage, with the partially removed thyroid plate indicated by the black arrow, the internal carotid artery shown by the white arrow, and the internal jugular vein shown by the blue arrow. (B) Removal of the posterior-lateral aspect of the thyroid cartilage, with the medial wall of the pyriform fossa indicated by the white arrow and the internal jugular vein shown by the blue arrow. (C) Opening of the pharyngeal lumen, with the remnants of the thyroid plate shown by the white arrow. (D) Complete resection of the tumor under direct visualization, with the posterior wall of the pharynx indicated by the white arrow. (E) Resected tumor specimen. (F) Pharyngeal mucosa and prevertebral fascia sutured for pharyngeal shaping; the black arrow indicates the mucosa of the pyriform fossa after tumor resection, while the white arrow indicates the laryngeal inlet. (G) Closure of the pharyngeal lumen; the black arrow points to the mucosa of the thyroid cartilage plate, and the white arrow shows the sutured pharyngeal lateral wall. (H) Freeing the submandibular gland and reinforcing the pharyngeal lateral wall incision; the black arrow points to the submandibular gland, the white arrow points to the bandeau muscle, and the blue arrow shows the internal jugular vein. (I) Submandibular gland fixed after suturing; the white arrow points to the strap muscles, the black arrow shows the submandibular gland after suturing, the blue arrow shows the internal jugular vein, and the yellow arrow indicates the common carotid artery.

**Figure 2.**
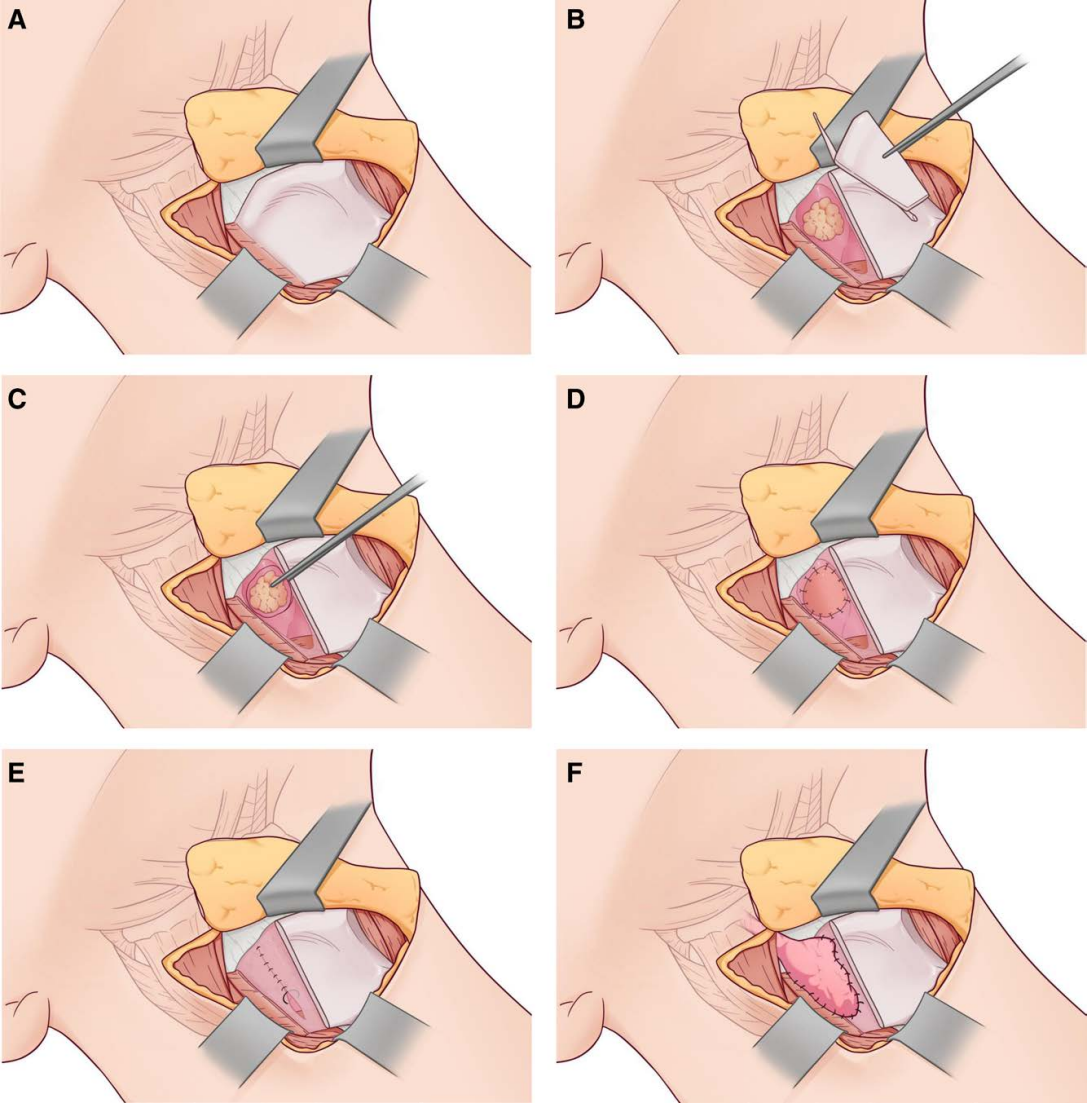
(A) Shown exposing the affected thyroid cartilage figure (red arrow); (B) Red arrow showing partial removal of the thyroid cartilage plate, black arrow showing the posterior pharyngeal wall tumor; (C) Complete resection of the tumor under direct visualization; (D) Pharyngeal mucosa and prevertebral fascia sutures for pharyngeal shaping; (E) Closure of the pharyngeal lumen with the sutured lateral pharyngeal wall shown by the black arrows; (F) Free submandibular gland underdrawn to reinforce the incision of the lateral pharyngeal wall with the submandibular gland demonstrated by the black arrows.

All patients underwent surgery under general anesthesia with tracheal intubation. They were placed in a supine position with shoulder pads, and the skin of the neck was sterilized. A low tracheotomy was performed under local anesthesia, the tracheal tube was inserted and secured. An anterior cervical “U”-shaped incision was made, which was extended in the middle portion. Functional cervical lymphadenectomy was carried out according to the TNM staging of the tumor and preoperative imaging evaluation. Subplatysmatic dissection was performed along the white line of the neck (Fig. 1A). The posterior-lateral portion of the thyroid cartilage on the affected side was resected (Fig. 2A and B), and the superior laryngeal artery was ligated on the same side. The superior horn of the thyroid cartilage was excised (Fig. 1B), and the hypopharyngeal constrictor muscle was detached from the thyroid cartilage membrane. The mucosa of the pyriform fossa was separated from the dorsal aspect of the thyroid cartilage (Fig. 1C). The mucosa of the pyriform fossa was then incised to access the pharyngeal cavity, allowing for complete resection of the tumor from the posterior hypopharyngeal wall under direct visualization (Figs. 1D, E and 2C). Rapid histopathological examination confirmed negative margins. The mucosa of the posterior hypopharyngeal wall was sutured and fixed to the prevertebral fascia (Figs. 1F, G and 2D). The mucosal defect was left open, and the mucosa on the pharyngeal side was sutured (Fig. 2E). The pharyngeal cavity was closed, and the submandibular gland on the affected side was freed, pulled down, and fixed (Figs. 1H and 2F). The upper and lower hyoid muscle groups were sutured, the strap muscles were sutured in counterpoint, and the cervical contour muscles, subcutaneous tissue, and skin were sutured in layers (Fig. 1I). A negative-pressure drainage tube was placed, a pressure bandage was applied, and antibiotics were administered to prevent infection in the postoperative period.

### 2.3. Postoperative observation and follow-up

Postoperatively, we monitored for complications including incision infection, bleeding, pharyngeal fistula, wound dehiscence, lung infection, and other issues. The timing of gastrostomy tube removal was recorded to assess the patients’ swallowing function. Initially, gastrostomy tubes were removed when patients showed recovery and could tolerate an oral liquid diet without significant choking. Follow-up assessments were performed using the Kubota water swallow test (WST)^[6]^ as the standard for evaluating swallowing function. In this test, patients were asked to drink 30 mL of warm water while sitting quietly, and both the time taken and any choking response were recorded. Swallowing function was categorized into 5 levels: Grade I, normal swallowing; Grade II, mild swallowing dysfunction; Grades III and IV, moderate swallowing dysfunction; and Grade V, severe dysphagia. Postoperative respiratory function was assessed by recording the time of tracheal tube extubation. For patients who did not require postoperative radiotherapy, extubation occurred once sufficient space in the pharyngeal cavity was observed using an electronic laryngoscope during a follow-up visit. For those who required radiotherapy, the tracheal tube was retained until the end of the radiotherapy course, at which point extubation was performed after confirming sufficient space in the laryngeal cavity with an electronic laryngoscope and ensuring normal breathing. After 48 hours of tracheal tube plugging, extubation was performed if no respiratory difficulty was observed.

Patients were instructed to attend outpatient follow-ups once a month during the first 3 months after surgery. After 3 months, follow-up intervals could be extended as appropriate. These reviews included examination of the pharyngeal lumen with an electronic laryngoscope to check for pharyngeal stenosis, and, if necessary, cervicothoracic enhanced CT scans were performed to assess for any distant recurrence or metastasis of the tumor.

## 3. Results

All 40 patients achieved stage I healing of their postoperative incisions without the formation of pharyngeal fistulas. The gastric tube was successfully removed prior to discharge, with a mean extubation time of 11 days. One month after surgery, 24 patients (24/40) had a WST grade of I, 12 patients (12/40) had grade II, and 4 patients (4/40) had grade III. By 3 months postoperatively, all 40 patients (40/40) had a WST grade of I, and their swallowing function had normalized. Tracheostomies were performed on all patients during the operation. Six patients were successfully extubated at 3 months due to delayed healing resulting from postoperative radiotherapy, while the remaining 34 patients were extubated within the expected time frame, with an average extubation time of 2 months. Since the tumor did not involve the vocal cords prior to surgery, none of the patients exhibited vocal abnormalities after the procedure. The 40 patients were followed for an average of 28 months (range: 12–48 months), with the most recent follow-up on January 31, 2024. None of the patients experienced tumor recurrence or distant metastasis.

## 4. Discussion

Carcinoma of the posterior wall of the hypopharynx is clinically rare, with most cases being squamous cell carcinomas that are highly malignant and poorly differentiated. According to an epidemiological study, approximately 91% of patients have a long history of smoking, and more than half have a history of alcoholism.^[7]^ Treatment options for posterior hypopharyngeal wall cancer include surgery, radiotherapy, chemotherapy, and biotherapy. Due to the proximity of the posterior hypopharyngeal wall lesion to the cervical spinal cord, the effectiveness of radiation therapy is limited. As a result, a comprehensive treatment approach, primarily based on surgery, remains the preferred choice for managing posterior hypopharyngeal wall cancer.^[8]^ With advances in medical knowledge and surgical techniques, the surgical treatment of posterior hypopharyngeal wall cancer is increasingly focused on functional outcomes. The goal is to provide a wide surgical field while ensuring complete tumor resection and minimizing damage to surrounding healthy tissues, thereby improving patients’ quality of life post-surgery.

An analysis of the clinical data from 16,248 cases of hypopharyngeal cancer in the U.S. National Cancer Data Bank over the past 20 years showed that nonsurgical treatment modalities, including radiotherapy, chemotherapy, concurrent chemoradiotherapy (CCRT), targeted therapy, and immunotherapy, have been incorporated into the comprehensive treatment of hypopharyngeal cancer. These approaches have improved laryngeal function preservation; however, the 5-year survival rate remains under 35%.^[9]^ Induction chemotherapy, administered before surgery or radiotherapy, aims to reduce the clinical stage of the tumor, decrease micro-infiltration, increase the chances of successful surgery or radiotherapy, improve laryngeal retention, and reduce tumor recurrence and distant metastasis. Commonly used induction chemotherapy regimens include PF (cisplatin + fluorouracil), TPF (docetaxel + cisplatin + fluorouracil), and PCE (paclitaxel + carboplatin + cetuximab).^[10]^ Synchronized radiotherapy is another crucial treatment option for hypopharyngeal cancer, significantly improving patient survival. A study comparing the efficacy of CCRT to radiotherapy alone found that patients in the CCRT group had a higher survival rate (63.5% vs 55.6%).^[11]^ Targeted therapy involves designing drugs to specifically target known cancer-causing sites at the molecular level, leading to selective tumor cell death with minimal cytotoxicity. Epidermal growth factor receptor is overexpressed in 80% to 90% of hypopharyngeal carcinomas and has been identified as both an oncogene and a therapeutic target.^[12]^ Cetuximab, the first epidermal growth factor receptor-targeted drug approved for hypopharyngeal cancer, has demonstrated promising results, with improved prognosis when added to treatment regimens.^[13]^ Janoray et al compared the efficacy of radiotherapy + cetuximab versus radiotherapy + cisplatin after induction chemotherapy with the TPF regimen for hypopharyngeal cancer. The study found no statistically significant difference in efficacy or adverse effects between the cetuximab and cisplatin groups, and no increase in adverse effects was observed.^[14]^ Immunotherapy is a therapeutic approach that controls and eliminates tumor cells by blocking the inhibition exerted by tumors on the immune system, regulating the immune microenvironment, and restoring the body’s normal immune response. This method relies on the body’s own immune function and has become a prominent area of cancer treatment research.^[15]^ In recent years, inhibitors targeting programmed death-1 (PD-1) and its ligand (PD-L1) have emerged as some of the most widely used immunotherapeutic agents in clinical practice, demonstrating significant efficacy in treating various types of cancer, including hypopharyngeal cancer. Ongoing clinical trials are also exploring the combination of immunotherapy with radiotherapy, chemotherapy, and other therapeutic modalities to achieve enhanced treatment outcomes. The success of immunotherapy in the clinical treatment of hypopharyngeal cancer offers new hope for patients with this condition.^[16]^

In recent years, induction chemotherapy and concurrent radiotherapy aimed at preserving laryngeal function have become increasingly common in the comprehensive treatment of hypopharyngeal carcinoma. However, large-scale studies have shown that the overall survival rate for hypopharyngeal carcinoma has not significantly improved. In contrast, surgery-based comprehensive treatment plans demonstrate greater advantages in terms of local tumor control and progression-free survival.^[17]^ The choice of surgical approach, the extent of resection, and the possibility of preserving laryngeal function primarily depend on the location, size, growth pattern, and infiltration of the primary tumor in the posterior wall of the hypopharynx.^[18]^ Studies have shown that T1, T2, and some T3 stage cancers of the posterior wall of the hypopharynx rarely invade the larynx. Since the larynx is situated anterior to the hypopharyngeal posterior wall and is at a considerable distance from the tumor, it is typically unaffected or only locally involved in cases of hypopharyngeal posterior wall cancer. This provides a pathological basis for the potential preservation of laryngeal function during surgery for hypopharyngeal posterior wall cancers. Additionally, advances in head and neck tumor reconstruction offer further possibilities for preserving laryngeal function.^[19]^ According to Yan Dangui et al,^[20]^ early-stage T1 and T2 lesions confined to the posterior wall of the hypopharynx, located above the esophagus and below the tip of the epiglottis, can preserve all laryngeal functions. Moreover, T3 lesions without laryngeal involvement also allow for full preservation of laryngeal function. The posterior hypopharyngeal wall cancer rarely invades the prevertebral fascia, which is considered safe from a pathological standpoint. Furthermore, the fascia is tough and resistant to infection, making it less susceptible to infection and necrosis. Yue et al^[21]^ reported the use of an anterior cervical approach with a posterior pharyngeal wall flap to repair defects after surgical resection of early-stage posterior hypopharyngeal wall cancer in 3 patients. These patients experienced good recovery of articulation, swallowing, and respiratory function postoperatively, achieving ideal results. However, the surgical trauma was still considerable. Xu et al^[22]^ conducted a study on 109 patients with squamous cell carcinoma of the posterior wall of the hypopharynx, using various surgical modalities. The results showed no significant difference in survival rates between different approaches. This suggests that, as long as the appropriate surgical access is chosen to ensure complete tumor resection, laryngeal function can be preserved, and pharyngeal function can be restored, ultimately improving the postoperative quality of life for patients. Lv Z et al^[3]^ examined 88 cases of hypopharyngeal posterior wall cancer and found that patients with N2 to 3 stage lesions had a high rate of retropharyngeal lymph node metastasis. They recommended routine exploration and clearance of retropharyngeal lymph nodes, noting that distant metastases were the primary cause of death in these patients. Canis et al^[23]^ highlighted the use of transoral microlaser surgery for treating posterior hypopharyngeal wall cancer, which can lead to a favorable prognosis. However, due to its narrow indication range and the high skill requirements for the surgeon, this technique limits its widespread application in clinical practice for posterior hypopharyngeal wall cancer. In this study, all 40 patients who underwent surgical treatment retained their laryngeal function. Through the lateral pharyngeal approach, the upper and lower extremities of the tumor could be fully exposed, and the adjacent relationship between the tumor and surrounding tissues could be clearly observed. After complete tumor resection, the repair was carried out based on the size of the tissue defect on the posterior pharyngeal wall. Among the 28 patients, the tissue defect was relatively small after tumor resection, and the residual mucosa was isolated and sutured to close the wound. For the 12 patients with a larger defect that could not be directly sutured by pulling and joining the surrounding mucosa, the residual mucosa of the lower pharyngeal posterior wall was sutured with the anterior spinal fascia. All 40 patients had their affected mandibular glands separated and repaired after closing the pharyngeal cavity, and achieved good results after surgery.

The presence of tumor remnants at the surgical margins and cervical lymph node metastases are key factors influencing survival and prognosis. According to Pan Xinliang et al,^[24]^ the resection of posterior hypopharyngeal wall cancer should ensure a safety margin of at least 1.5 cm around the tumor, with the upper and lower edges of the tumor margin extending 2 cm, while Hayashi et al^[25]^ recommended a minimum of 5 mm for the deeper part of the tumor margin. In this study, tumor resection was performed based on these margins, and intraoperative frozen pathological examination of the upper, lower, left, and deep margins was conducted, with negative results confirming complete resection. Due to the high density of lymphatic vessels in the posterior hypopharyngeal wall area, lymph node metastasis is common, with the most frequent metastasis occurring in the ipsilateral cervical lymph nodes at levels II and III.^[26]^ A poor prognosis is suggested when there is involvement of other regional lymph nodes, bilateral cervical lymph nodes, or even paratracheal and retropharyngeal lymph nodes. Pharyngeal fistula and dysphagia are the most common complications following surgery for posterior hypopharyngeal wall cancer. Dysphagia mainly results from inadequate space in the reconstructed hypopharyngeal cavity or the formation of scar tissue, causing obstruction during swallowing and potentially blocking the laryngeal opening, leading to aspiration. Pharyngeal leakage primarily occurs due to intraoperative damage to the pharyngeal wall’s vascular network, causing ischemia, bruising, and localized necrosis of tissues. Additionally, a dead space in the reconstructed hypopharyngeal mucosa, coupled with the absence of smooth negative-pressure drainage, contributes significantly to pharyngeal leakage. In this study, the tissue defects on the lateral side of the pharyngeal anastomosis were repaired with a free submandibular gland, and the carotid artery was wrapped and sutured with part of the sternocleidomastoid muscle after neck dissection to isolate the carotid artery from the hypopharyngeal anastomosis. Negative-pressure drainage and a pressure bandage were applied to effectively prevent the formation of pharyngeal leakage.

## 5. Conclusion

In summary, this study demonstrates that the lateral pharyngeal approach for the resection of posterior hypopharyngeal wall cancer offers several advantages. Regarding tumor resection, this approach enables comprehensive exposure of the tumor’s upper and lower poles, allowing for safe and precise excision under direct visualization. In terms of functional preservation, it minimizes damage to surrounding tissues, facilitating effective recovery of swallowing, respiratory, and speech functions. For tissue repair, the approach allows for tailored reconstruction strategies based on the defect, effectively preventing pharyngeal fistulas and other complications through specific surgical techniques. However, this study has certain limitations. The lateral pharyngeal approach alone is challenging for T3 tumors involving the larynx or T4 tumors, necessitating a combined anterior cervical approach. Additionally, the small sample size and lack of a control group (with no comparative efficacy data against other surgical or nonsurgical methods) precluded formal statistical analysis. Although we observed reduced patient trauma, tissue loss, and complication rates, these findings may not be broadly generalizable and could be influenced by various confounding factors.

To address these limitations, future research should incorporate a larger sample size, multicenter settings, and a prospective study design, including a control group, to enable more rigorous statistical evaluation. Such studies would provide a more accurate assessment of the efficacy of lateral pharyngeal access surgery and facilitate comparisons with existing treatment modalities. Despite these constraints, our study offers valuable preliminary insights into the application of the lateral pharyngeal approach for treating malignant tumors of the posterior hypopharyngeal wall. It also establishes a foundation for future research and clinical practice in this domain. These outstanding results were obtained in a highly selected T1–T3 cohort treated by a single surgical team; they should be regarded as promising preliminary findings that mandate validation through larger, multi-center prospective studies before the technique is widely adopted.

## Author contributions

**Conceptualization:** Qingyong Chen, Zhipeng Chen, Qiang Shao, Huaiqing Lv, Liqiang Lin.

**Data curation:** Qingyong Chen, Xiaoli Liu, Zhipeng Chen, Huaiqing Lv, Liqiang Lin.

**Formal analysis:** Qingyong Chen, Xiaoli Liu, Qiang Shao.

**Investigation:** Qingyong Chen, Zhipeng Chen, Liqiang Lin.

**Methodology:** Qingyong Chen, Xiaoli Liu, Qiang Shao.

**Project administration:** Liqiang Lin.

**Resources:** Qingyong Chen, Xiaoli Liu, Liqiang Lin.

**Software:** Qingyong Chen, Xiaoli Liu, Zhipeng Chen, Huaiqing Lv.

**Supervision:** Qingyong Chen, Qiang Shao, Huaiqing Lv, Liqiang Lin.

**Validation:** Zhipeng Chen, Qiang Shao, Liqiang Lin.

**Visualization:** Xiaoli Liu, Liqiang Lin.

**Writing – original draft:** Qingyong Chen, Qiang Shao, Huaiqing Lv, Liqiang Lin.

**Writing – review & editing:** Qingyong Chen, Zhipeng Chen, Liqiang Lin.
